# Circ-camk4 involved in cerebral ischemia/reperfusion induced neuronal injury

**DOI:** 10.1038/s41598-020-63686-1

**Published:** 2020-04-24

**Authors:** Zhao-huan Zhang, Yue-rong Wang, Fei Li, Xiu-ling Liu, Hui Zhang, Zhong-zheng Zhu, Hai Huang, Xiao-hui Xu

**Affiliations:** 1 School of Preclinical Medicine, Wannan Medical College, Wuhu 241001, China. School of Life Sciences, Shanghai University, Shanghai, 200444 China; 2 Department of Neurology, Changzheng Hospital, 200003 Shanghai, China; 3 School of Life Sciences, Shanghai University, Shanghai, 200444 China; 4 Department of Oncology, Shanghai Tenth People’s Hospital, Tongji University School of Medicine, Shanghai, 200072 China

**Keywords:** Stroke, Neurological disorders

## Abstract

Stroke and subsequent cerebral ischemia/reperfusion (I/R) injury is a frequently occurring disease that can have serious consequences in the absence of timely intervention. Circular RNAs (circRNAs) in association with microRNAs (miRNAs) and RNA-binding proteins (RBPs) can influence gene expression. However, whether circRNAs have a role in cerebral I/R injury pathogenesis, especially soon after onset, is unclear. In this study, we used the SD rat middle cerebral artery occlusion (MCAO) model of stroke to examine the role of circRNAs in cerebral I/R injury. We used high-throughput sequencing (HTS) to compare the expression levels of circRNAs in cerebral cortex tissue from MCAO rats during the occlusion-reperfusion latency period 3 hours after I/R injury with those in control cerebral cortices. Our sequencing results revealed that expression levels of 44 circRNAs were significantly altered after I/R, with 16 and 28 circRNAs showing significant up- and down-regulation, respectively, relative to levels in control cortex. We extended these results *in vitro* in primary cultured neuron cells exposed to oxygen-glucose deprivation/reperfusion (OGD/R) using qRT-PCR to show that levels of circ-camk4 were increased in OGD/R neurons relative to control neurons. Bioinformatics analyses predicted that several miRNAs could be associated with circ-camk4 and this prediction was confirmed in a RNA pull-down assay. KEGG analysis to predict pathways that involve circ-camk4 included the glutamatergic synapse pathway, MAPK signaling pathway, and apoptosis signaling pathways, all of which are known to be involved in brain injury after I/R. Our results also demonstrate that levels of the human homolog to circ-camk4 (hsa-circ-camk4) are elevated in SH-SY5Y cells exposed to OGD/R treatment. Overexpression of hsa-circ-camk4 in SH-SY5Y cells significantly increased the rate of cell death after OGD/R, suggesting that circ-camk4 may play a key role in progression of cerebral I/R injury.

## Introduction

For decades, stroke has been one of the most emergent causes of mortality and disability for adults.The majority of stroke cases involveischemic stroke, whereinblood flow in the brainis interrupted by a clot. This interruptionleads to oxygen and nutrient depletion that cause neuronal tissue death in the injury core. Secondary injury is initiateddue to the accumulation of reactive oxygen species (ROS) and toxicity of excitatoryamino acids^1^. ROS and excitatory amino acids promote inflammation and activation of multiple cell-signaling responses that in turn lead to apoptosis and necrosis. Restoration of blood perfusion following an ischemic stroke can sometimes further extend and aggravate areas of ischemic injuries, a condition termed ischemia/reperfusion (I/R) injury. Brain tissue is particularly sensitive to I/R^2^, and timely treatment of I/R injury is critical to avoid serious sequelaeassociated with neuronalcell death^3^. Therefore, an understanding of mechanisms that are invoked early after I/R injury is needed to understand the pathogenesis of stroke.

Non-coding RNAs (ncRNA) including transport RNA (tRNA), ribosome RNA (rRNA), small nucleolar RNA (snoRNAs), small nuclear RNA (snRNA), and microRNA (miRNA) are the most abundant genomic transcriptsand play a variety of functions beyond protein translation. Mature miRNAs are typically short ncRNAs (20–24 nt) and are universal post-transcriptional regulators of gene expression in most multicellular organisms. miRNAs also play roles in fundamental cellular functions and are implicated in both physical processes and disease development. Primary transcripts of miRNA (pri-miRNAs) areprocessed by the Droshari bonuclease III to produce precursor miRNA (pre-miRNA) with an approximately 70-nt stem-loop that is further processed by cytoplasmic Dicer ribonuclease to generate mature miRNA. Mature miRNAs associate with a RNA-induced silencing complex (RISC) to recognize target mRNA viabase paring that consequently results in target mRNA transcription inhibition or degradation. This base pairing is imperfect such that one miRNAcan target multiple genes and one gene can be recognized by multiple miRNAs, which adds to the complexity ofpost-translation regulation of gene expression.

Previous studies proposed that transient focal ischemia could causechanges in miRNA expression in both the brain and blood vessels^4^. Bioinformatics analysis showed that miRNAs can indeed target different genes to affect protein translation after stroke and regulate excitotoxicity, inflammation, autophagy, apoptosis and oxidative stress. Previous studies established a role for miRNAs in post-I/R neuronal apoptosis, which is attributed to ischemia-induced neuron injury^5^.

Recently, the family of ncRNAs expanded to include circular RNAs (circRNAs), which feature a circular transcript arising from head-to-tail backsplicing. Given that such circRNAs possess neither a 5′ nor a 3′ end, circular RNAs are naturally resistant to digestion by RNaseR. circRNAs constitute a substantial fraction of the mammalian transcriptome and are abundantly expressed in the brain^6^. Multiple functional studies revealed that circRNAs act as miRNA “sponges” that regulate gene expression by sequestering miRNAs and preventing interactions with target mRNAs, including linear transcripts of parental genes^7^. For instance, the miR-7 inhibitor circRNA ciRS-7 (or CDR1as) contains at least 70 conventional miR-7-binding sites^8^. The functional role of circRNAs in the brain is now emerging. Recent evidence in the mouse middle cerebral artery occlusion (MCAO) model demonstrated that circular RNA are involved in neuronal cell injury after I/R^9^. Moreover, circular RNA Hectd1 (circHectd1) levels were found to be significantly increased in ischemic brain tissues in the mouse model of stroke induced by transient MCAO (tMCAO). Knockdown of circHectd1 expression promoted activity of endogenous miR-142 and significantly decreased infarct areas in tMCAO mice^10^. However, the miRNA targets of only a few circRNAs have been clearly defined and the expression patterns, expression levels and physiological roles of most circRNAs remain unknown. In particular, the role of circRNA during the early stages after stroke is unclear.

In this study, we sequenced the total circular RNA from the focal ischemia side and control side in a MCAO rat model during the very early stages after injury to identify candidate circRNAs that may be involved in I/R injury. Sequence analysis showed that these circRNAs are highly abundant in the ischemia cortex, although their definite biological function was unclear. We further characterized one abundant circRNA, circ-camk4, a circRNA transcribed from the exons 8, 9 and 10 of the rat calcium/calmodulin-dependent protein kinase 4 (camk4) gene. Bioinformatics analysis revealed that multiple miRNAs could bind to circ-camk4, and we further confirmed some of the genes that are targeted by these mRNAs. Finally, we found that overexpression of circ-camk4 could promote cell death *in vitro*, indicating that circ-camk4 might be involved in the pathological progression following brain stroke injury and could be apromising new molecular target for clinical therapy.

## Materials and methods

### Middle cerebral artery occlusion model

MCAO model surgical procedures on rats were approved by the Research Animal Resources and Care Committee of Shanghai University. Focal ischemia was induced via middle cerebral artery occlusion (MCAO) in male Sprague Dawley (SD) rats following a previously described procedure^11^. Briefly, male Sprague Dawley rats weighing around 200 g were anesthetized by inhalation of 1.5% isoflurane and kept in a position in which the neck was exposed. The neck skin was cut from the midline and draw hooks were used to maintain full exposure of the operative field. The left internal and external carotid arteries were dissected from the surrounding tissues, and a silicon-coated 4-0 nylon thread was inserted into the internal carotid artery to a depth of 18 ± 0.5 mm to occlude the base of the left middle cerebral artery. Transient focal cerebral ischemia was induced for 1 hour after which the nylon thread was retracted and the incision was sutured. After 3 hours of reperfusion, the brains were removed and 8–11 mm-thick and 2–3 mm diameter slices from the frontal pole (bregma −2.0 to −5.0 mm ± 1 mm) to the neocortex of both sides (MCAO side and the contralateral intact side) at 2 o’clock were dissected. The slices were snap-frozen and stored at −80 °C until further HTS analyses or RT-qPCR determination.

### Primary neuronal cell culture and OGD/R model

Postnatal day 1 (P1) Sprague-Dawley rats were used for isolation of primary neuronal cells as previously described^12^. The rat primary cortical neurons were cultured in Neurobasal + B27 medium (Invitrogen) in a normoxic (21% O_2_, 74% N_2_ and 5% CO_2_) cell culture incubator at 37 °C. OGD/R neuron cells were produced for use in an *in vitro* model of cerebral I/R. Briefly, neuron cells were cultured in glucose-free MEM and then placed in hypoxic conditions (1% O_2_, 94% N_2_ and 5% CO_2_) at 37 °C for 2 h or 4 h. The medium was then exchanged with Neurobasal + B27 medium and cultured for another 1 h or 2 h with reoxygenation (21% O_2_, 74% N_2_ and 5% CO_2_) to produce OGD/R 3 h and OGD/R 6 h cells, respectively. Cells not exposed to hypoxic conditions served as a control.

### Cell culture and transfection

C6 and SH-SY5Y cells were both kindly provided by Stem Cell Bank, Chinese Academy of Sciences. C6 cells cultured in F12K medium (Hyclone, USA) containing 15% horse serum (GIBICO, USA) and 2.5% fetal bovine serum (BI, USA), The SH-SY5Y cells was cultured in Dulbecco’s modified Eagle’s medium (DMEM, BI, USA) containing 10% fetal bovine serum (FBS, BI, USA) in a 37 °C incubator with a 5% CO_2_ humidified atmosphere For transfection, he cells were seeded 24 hrs before at a confluency of 40–80%, and the transfection were performed using Lipo3000 according to the manufactures’ guide.

### SH-SY5Y cell OGD/R model

SH-SY5Y cells were seeded at 80% confluency in 6-well plates (FALCON, Corning Inc, USA). After 24 hrs. of culture, cells were transfected with hsa-circ-camk4 over expression plasmid, and empty vector or miss-matched circ-camk4 as corresponding controls. Transfection were performed by using Lipofectamine 3000 (Invitrogen, Carlsbad, CA, USA) according to the manufacture’s guide. Forty-eight hours post transfection, medium were replaced with MEM, and cells were placed in hypoxic conditions (1% O_2_, 94% N2, 5% CO_2_) at 37 °C for 2 hrs (OGD/R-3h) or 4 h (OGD/R-6h). Thereafter, the medium was changed with DMEM culture medium and reoxygenation (21% O2, 74% N2 and 5% CO_2_) for 1 hr((OGD/R-3h)) or 2 hours ((OGD/R-6h). Other cells of unchanged condition served as a control. PI/Hoechst staining been used to detect the death cells.

### RNA isolation, quality control and library preparation for high throughput sequencing

Total RNA was extracted from the samples with TRIzol® Reagent (Invitrogen) according to the kit’s instructions. The total RNA concentrations and quality in the tissue samples were then assessed by NanoDrop2000 and Agilent 2100. Specifically, OD260/OD280 ratios between 1.8 and 2.2, and OD260/OD230 ratios of greater than 1.8 deemed acceptable, while RIN should be over 0.85. In order to enrich pure circRNAs, rRNAs and linear RNAs were removed from the total RNA in each sample by Ribo-Zero Magnetic kit and RNase R (Epicentre) treatment. RNA-seq library were preparared by using the TruSeq™ Stranded Total RNA Library Prep Kit (Illumina) following the manufacturer’s instructions. (LifeTechnologies,USA). TBS380 Picogreen (invitrogen) was adopted to quantified the libraries and Illumina Hiseq. 4000 were used for pair-end HTS. The library preparation and sequencing was performed at Major-Biotech Inc. (Shanghai, China).

### Bioinformatic analysis and miRNA target prediction

SeqPrep(https://github.com/jstjohn/SeqPrep)was used for quality control (QC) of primary RNA seq reads, which were trimmed using Sickle software(https://github.com/najoshi/sickle). The trimmed reads were mapped to the rat reference genome(rno6) using Bowtie (http://bowtie-bio.sourceforge.net/index.shtml) or Burrows-Wheeler Transform (BWT). Next, Known and Novel IsoForm Explorer (KNIFE; https://github.com/lindaszabo/KNIFE.) was used to predict circRNAs from sequence data. By annotating each pair of reads to the junction index, junctions having a posterior probability > 0.9 were considered to have a strong probability to be circular junctions, which were mostly annotated to cover the reverse splice junction site. Only circular-junction annotated reads, i.e., those reads mapping within the range of a same junction site (junction index) were used for further quantification and comparison of expression. EdgeR software was used to identify differentially expressed genes (DEGs) between MCAO and control libraries. Primary inclusion criteria for DEGs were |logFC | ≥1 and P value < 0.05. A more strict filter was then applied to down-select to identify candidates with |logFC | ≥ 1.5 where FC represents fold-change.

CircRNA/miRNA interactions were predicted with miRanda (http://www.miRNA.org/miRNA/home.do) and the top 4 putative target miRNAs were selected. The putative target genes of these miRNAs were identified using Target scan (Target Scan http://www.targetscan.org). A circRNA-miRNA-gene network was generated using Cytoscapeto visualize the interactions. GO and Kyoto Encyclopedia of Genes and Genomes (KEGG) pathway enrichment analysis together with Blast2Go and TopGO via NovelBrain BioCloud (NovelBio. Inc., Shanghai, China) were used to annotate target gene functions.

miRNA response elements (MREs) were predicted using RNAhybrid software with aminimal free energy (mfe) cut-off value of −20 kcal/mol.

### Quantitative real-time polymerase chain reaction (qRT-PCR) Analysis

Total RNA was extracted from brain tissue or primary cultured neurons as described above followed by DNase I treatment and then purified by miRNeasy® Mini Kit (Qiagen). The reverse transcription (RT) reaction 10 ul of 500 ng total RNA was performed with random primers using a PrimeScript RT Master Mix (Perfect Real Time) (TAKARA Cat.RR036A). Quantitative RT-PCR was performed using SYBR Green qPCR SuperMix (TAKARA Code No. RR420A). The PCR conditions were at 95 °C denaturation for 1 min, followed by 40 cycles at 95 °C for 10 s and 60 °C for 15 s. Melt curve analysis was carried out after the PCR to confirm primer specificity. The primers used for the qRT-PCR analysis as follows:

Rno-circ-camk4-Fwd: 5′-ATCGTGGAACATCAAGTGCTCA-3′

Rno-circ-camk4-Rev: 5′-AACTGCCTCCAGGATCTGCTT-3′

U6-Fwd: 5′-GGAACGATACAGAGAAGATTAGC-3′

U6-Rev: 5′-TGGAACGCTTCACGAATTTGCG-3′

Hsa-circ-camk4-Fwd: 5′-AATTGTGGAACATCAAGTGCTCA-3′

Hsa-circ-camk4-Rev: 5′-GCAACTGCCTCCAGGATTTGTT-3′

The relative expression of circRNAs was calculated using the 2^−ΔΔCt^ method.

### Vector construction

To transcript circRNA, the genomic region for hsa-circ-camk4 was amplified using Primer-F: CAGAAATTAATTAAGGATCCGATTGTGGAAAAGGGATATTACAGTG

Primer-R: GCCGGCCGGTAACCGAATTCCGCAGTACCCTGGGGTTC

The PCR products were inserted into GPLVX-Laccase2-R_circRNA-GFP-Puro vector (The Genomeditech Ltd, Shanghai, China). The miss-matched circ-camk4 is the has-circ-camk4 sequence reversed inserted into the GPLVX-Laccase2-R_circRNA-GFP-Puro vector.

### Biotinylated miRNA pull down assay

The 3′ ends of miRNAs of interest were labeled with biotin. Scrambled control miRNAs were commercially provided by Genepharma Co. (Shanghai, China).

RNA pull down assays were performed as previously reported^13,14^ with some modifications. Briefly, rat C6 cells were seeded in 10 cm tissue culture dishes at 30% confluency, and transfected 24 hours later with the 3′ biotin-labeled miRNAs or the scramble control miRNA at a final concentration of 50 nM using Lipofectamine 3000 (Thermo Fisher) according to the manufacturer’s recommendations. At 24 h after transfection, the cells were lysed with 550 µl mild lysis buffer. The whole cell lysates were centrifuged and 50 µl of the supernatant was mixed with 1 ml Trizol for RNA preparation as the input control, and another 500 µl was incubated with Dynabeads™ MyOne™ Streptavidin C1 (Invitrogen, USA) for 2 hr at 4 °C with gentle rotation. The beads were then washed 5 times with lysis buffer and reverse transcription was performed for qPCR determination.

### Statistical analysis

All data were analyzed GraphPad Prism 5.0 statistical software and are presented as the mean ± standard deviation (SD). Differences in the relative expression of circRNAs between MCAO (LI) and contralateral control (RC) samples as verified by qRT-PCR were analyzed by paired sample t-tests. A P value < 0.05 was considered statistically significant.

### Compliance with Ethical Standards

#### Research involving animals

All the animals in this study were obtained from Joint Ventures Sipper BK Experimental Animal (Shanghai, China). The animal experiments were undertaken in accordance with the National Institutes of Health Guide for the Care and Use of Laboratory Animals and with the approval of the Shanghai University Committee on Animal Care.

## Results

### CircRNA expression profiles are altered in the cerebral cortex after I/R

**Figure 1:**
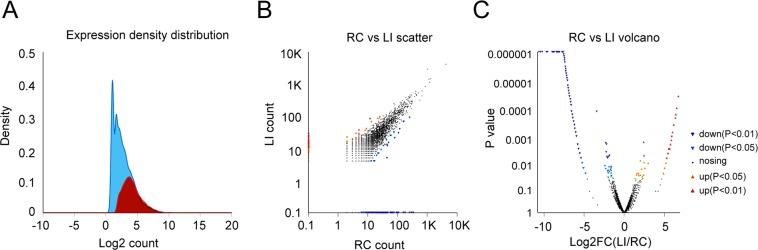
Differences in circRNA expression profiles between MCAO and control groups. The left cerebral cortex underwent MCAO(LI). The right cerebral cortex was subjected to the same operation without occlusion and served as the control group(RC). (**A**) Circular RNA expression quantification shows significantly different circRNA expression profiles between the two groups. (**B**) Scatter plots in which the ordinates and transverse ordinates represent circRNA expression (counts) for LI and RC, respectively. The values of both groups were processed logarithmically, and each point represents a specific circRNA. A specific point corresponding to the ordinate and transverse ordinate values indicates the expression level of a circRNA in the LI and RC, respectively. The red and blue points indicate significant up- and down-regulation, respectively, and the black points indicate absence of statistically significant differences. P value < 0.05 by Fisher exact test by a qCML method. (**C**) Volcano map (Volcano-plots). The transverse coordinates are the multiplier values of the fold-change (FC) between LI and RC. The ordinate is the statistical test value of the difference in the variation of the expression, indicated as P. The numerical value of the transverse ordinate was processed logarithmically. Each point represents a specific circRNA, with red and blue points representing significant up- and down-regulated circRNAs and black points indicating no significant change. P value < 0.05 by Fisher exact test.

We used high-throughput RNA sequencing to explore changes in rat circRNAs during the early onset of I/R in cortex tissue from rats with MCAO for 1 hr followed by 3 hr reperfusion (Left Injury, LI). Tissue from the untreated contralateral right side served as the control group (Right control, RC). After QC, a total of 112,317,640 and 107,426,430 clean reads were obtained for LI and RC, respectively. The data also had 15,071,637,200 and 14,380,514,607 bp for LI and RC, respectively (Error(%), 0.0102 vs. 0.0101; Q20 (%), 98.47 vs. 98.52; Q30 (%), 95.43 vs. 95.61, and GC composition(%), 59.49 vs. 59.26). The QC data showed equal depth and quality for the sequencing of both libraries. Given that individual circRNAs varied widely in size, despite the split junction, their sequences are the same as the linear transcript, such that longer transcripts will have more read counts. To increase accuracy, we used only those reads that spanned the junction for expression analysis. Here we used KNIFE analysis to identify circRNAs and simultaneously determine the counts that mapped to individual junctions. The number of total reads that annotated to circular junctions was 109,567 and 116,274 for LI and RC, respectively (Figure S1A,CIRC_SRTONG). Total junctions were identified and divided into three types: regular splicing junctions (Reg); reverse splicing junctions of more than 2 exons (Rev); and back splicing within a single exon (Dup). Here we annotated 33,829 and 28,692 junctions for LI and RC, respectively. Of these, 3,172 for LI and 8,117 for RC were circular RNA junctions comprising one or more exons. Moreover, 2,751 junctions were identified in both LI and RC (Figure S1C). Although the total counts were similar, the distribution pattern varied substantially (Fig. 1A). The expression pattern of the circRNA junctions for RC was more evenly distributed whereas that for LI tissue was much more concentrated. Moreover, the average counts at each junction was higher in LI tissue than in RC due to the identification of different circRNA junctions. These data indicated that marked post-ischemic changes could be observed in the levels of individual circRNAs. We thus used EdgR to identify differentially expressed genes (DEGs) between the two tissue types (Fig. 1B,C). A scatter-plot was used to represent the counts for LI and RC, and ordinate and transverse values were processed logarithmically (Fig. 1B). Volcano-plots were also used to show fold-change (FC) of the DEGs (Fig. 1C). The EdgeR analysis with default parameters identified 1,581significant DEGs (p < 0.05), of which 44 were present in both LI and RC and also had |logFC | ≥1.5. Among these 44, 16 circRNAs were significantly up-regulated and 28 were down-regulated. The log2 FC(LI/RC) of the 16 up-regulated circRNAs ranged between 1.536 and 3.008, while that for the 28 that were down-regulated ranged from −1.562 to −3.449 (Supplemental Table 1).

### Validation of post-ischemic changes in circRNA expression by RT-qPCR

**Figure 2:**
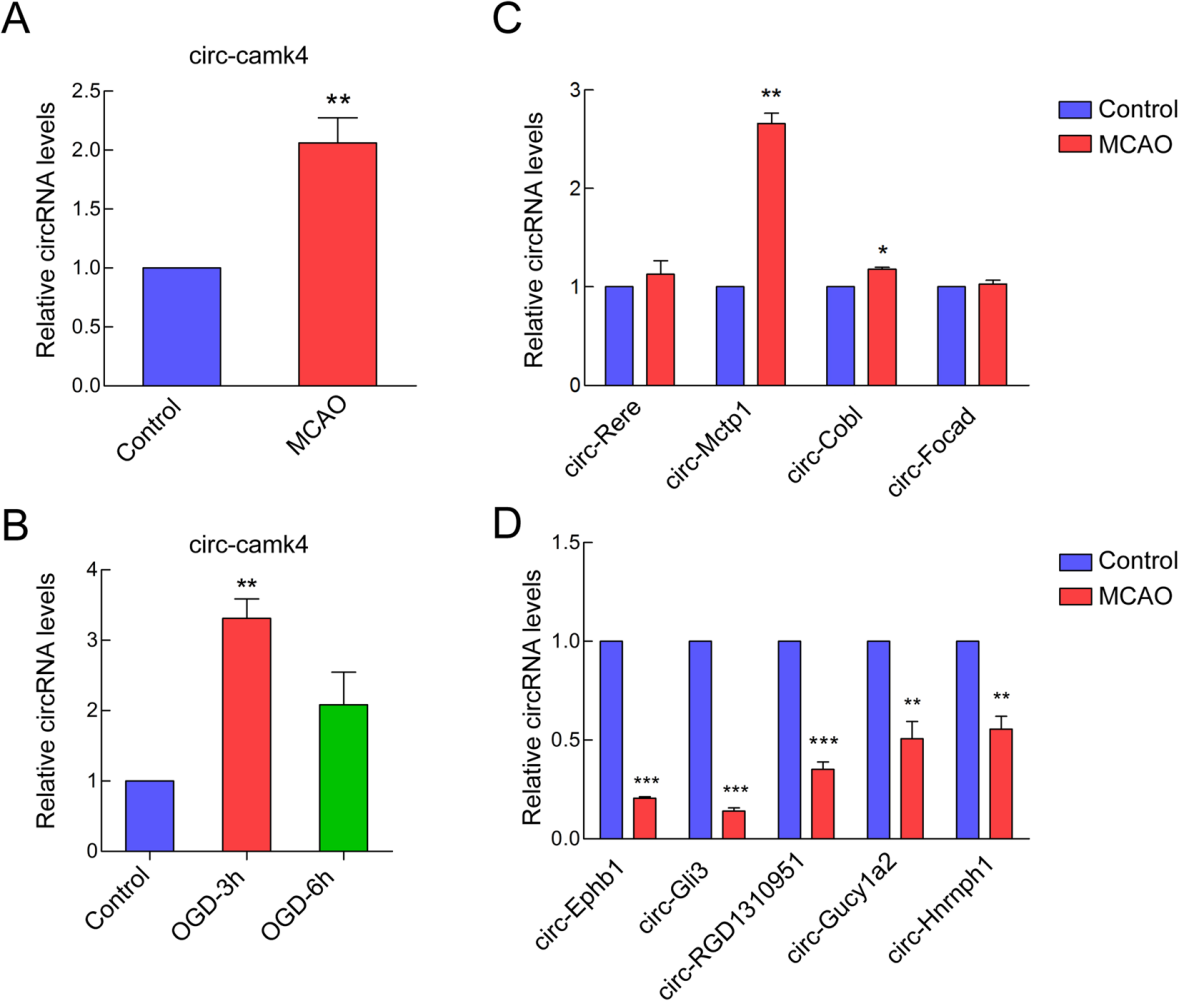
Circ-camk4 was more abundant in MCAO groups than in control groups. (**A**) qRT-PCR analysis was used to validate differential expression of circ-camk4 in the rat MACO model. (**B**) Circ-camk4 expression was up-regulated in the OGD/R-3h and 6 h groups compared to the control group. Differential expression of (**C**) 4 other up-regulated and (**D**) 5 down-regulated circRNAs shown by qRT-PCR. (Bars represent mean ± SD; ***p < 0.001, **p < 0.01, *p < 0.05 vs. control group).

We further verified changes in circ-camk4 by qRT-PCR. Based on the junction index annotation of the RNA sequencing data, the genomic locus of circ-camk4 is chromosome 8 with cyclization of exons 8, 9 and 10. The circ-camk4 expression levels were quantified by qRT-PCR with divergent primers calibrated using standard curves. The primary data were first standardized relative to GAPDH expression and then normalized with respect to the control. Consistent with the RNA-seq results, circ-camk4 expression was significantly increased by around 2-fold in MCAO samples(n = 4) relative to that seen for the control group samples (n = 4) (Fig. 2A). We also used primary cultured neuronal cells exposed to OGD/R as an *in vitro* model to test whether circ-camk4 expression in neuronal cells responded to glucose/oxygen depletion for 3 hr or 6 hr and subsequent recovery. Expression of circ-camk4 was up-regulated by at least 2-fold in both the OGD/R-3h and OGD/R-6h groups compared to the control group (Fig. 2B).We also verified another 4up-regulated circRNAs(Figs. 2C and 5) that were down-regulated circular RNAsby qRT-PCR (Fig. 2D).

**Figure 3:**
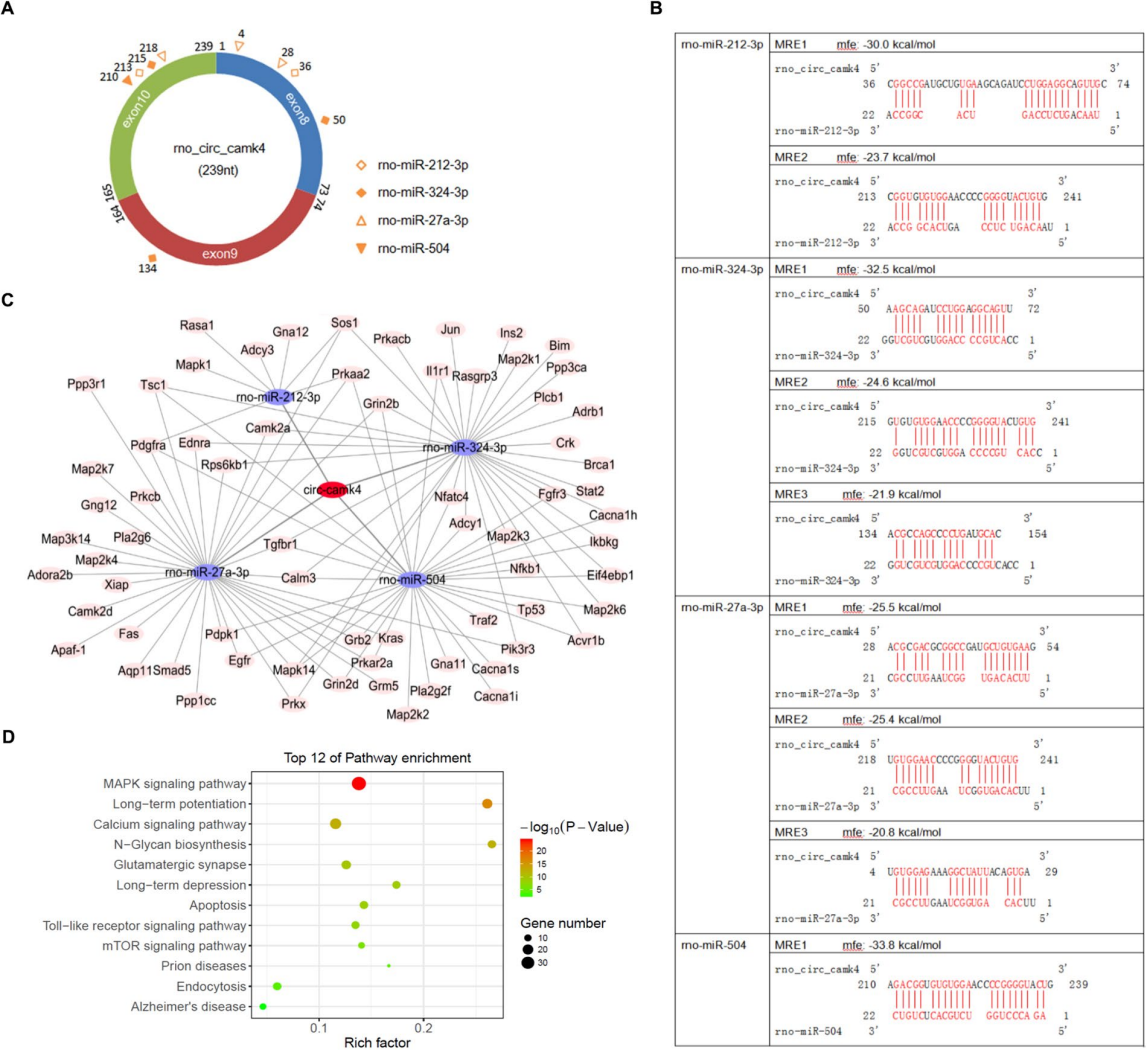
Bioinformatic analysis of circ-camk4 function. The circ-camk4-miRNA-gene network was predicted using Targetscan and miRanda. The top four predicted miRNA targets of circ-camk4 were: rno-miR-27a, rno-miR-324-3p, rno-miR-212 and rno-miR-504. (**A**) Schematic diagram of binding sites for the top 4 miRNAs on circ-camk4 CDS as predicted by RNAhybrid software. (**B**) Individual MREs (mfe < -20 kcal/mol) analyzed by sequence alignment. (**C**) Schematic diagram of circ-camk4-miRNA-gene network constructed using Cytoscape software and showing predicted functional connections between circ-camk4 (red nodes), target miRNAs (blue nodes) and downstream regulated genes (orange nodes) in the network. (**D**) Cell signaling pathway annotations by KEGG analysis for genes targeted by miRNAs associated with circ-camk4. The enrichment factor represents the ratio of enriched differential genes to annotated genes in each pathway. The area of each node represents the number of enriched differentially expressed genes. The p-values are indicated by different colors with green indicating high and red indicating low values.

**Figure 4:**
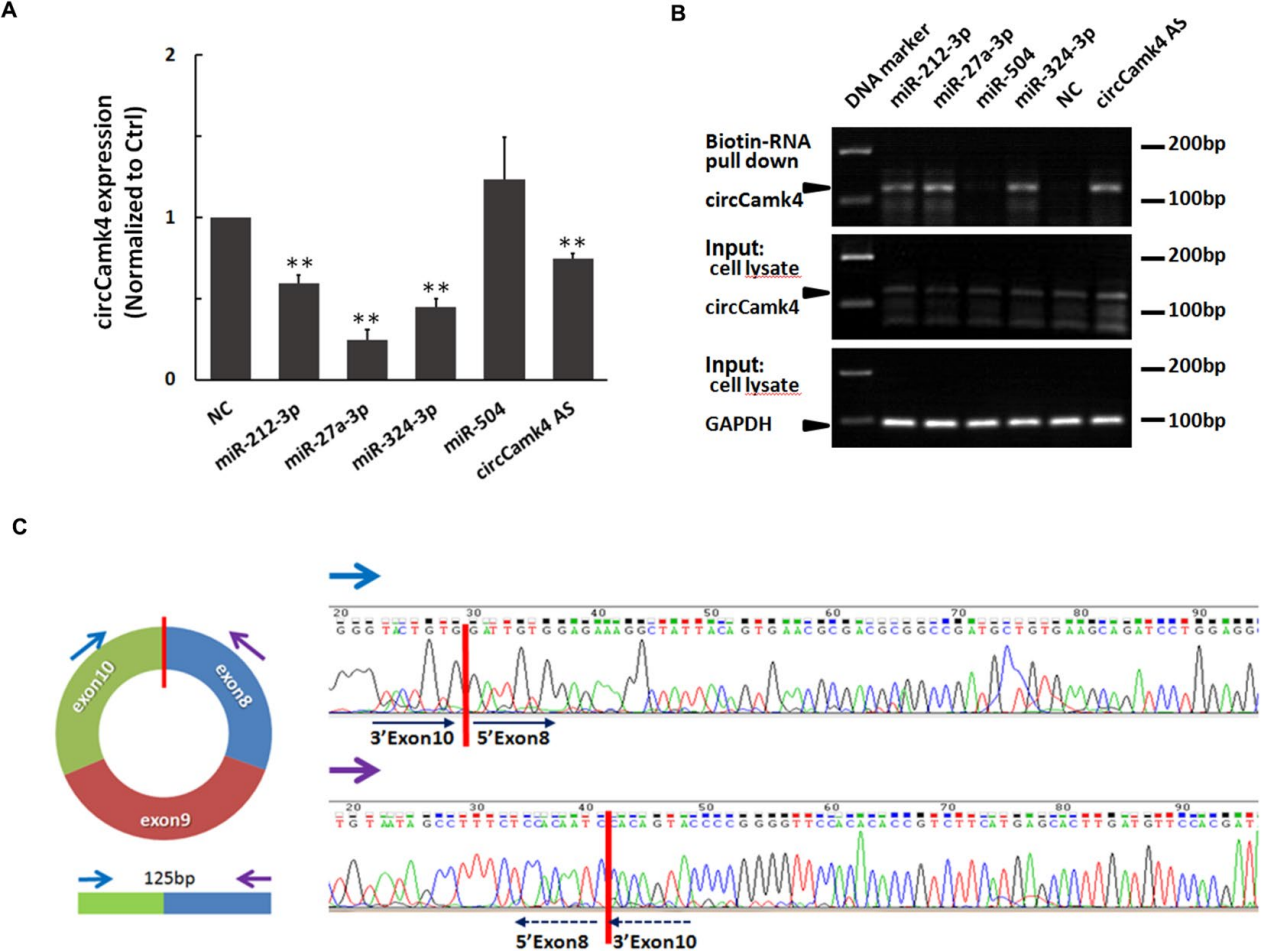
Confirmation of circ-camk4 target miRNA. C6 cells were harvested 24 hr post-transfection with 50 nM biotinylated miRNA mimics (rno-miR-212-3p, rno-miR-27a-3p, rno-miR-212-3p and rno-miR-504), a biotinylated antisense RNA probe for circCamk4 (circCamk4 AS) or a non-targeting control RNA (NC). (**A**) Endogenous circCamk4 expression detected by qRT-PCR 24 hr after miRNA transfection. CircCamk4 expression was first standardized relative to GAPDH input and the relative expression levels were compared using Student’s *t*-test, **p < 0.01, ***p < 0.001. (**B**) RNA pull-down assay to identify miRNA candidates that target circCamk4. Biotinylated RNAs and associated species were purified with streptavidin beads, following by qRT-PCR quantification of circCamk4 expression. RNA templates from individual whole cell lysates were used as input controls. Abbreviations: circCamk4 AS, circCamk4 antisense probe; GAPDH, glyceraldehyde 3-phosphate dehydrogenase; NC, non-targeting control miRNA. (**C**) Schematic diagram showing the divergent primer used for circCamk4 qPCR that spans the splice junction of exon 10 and exon 8, with a 125 bp amplicon. Red lines highlight regions that were Sanger-sequenced and identified as the splice junction of circ-Camk4. Blue and purple arrows indicate divergent primers. Black arrows show the direction of duplication by PCR in Camk4 CDS sequencing with the divergent primer pair.

**Figure 5:**
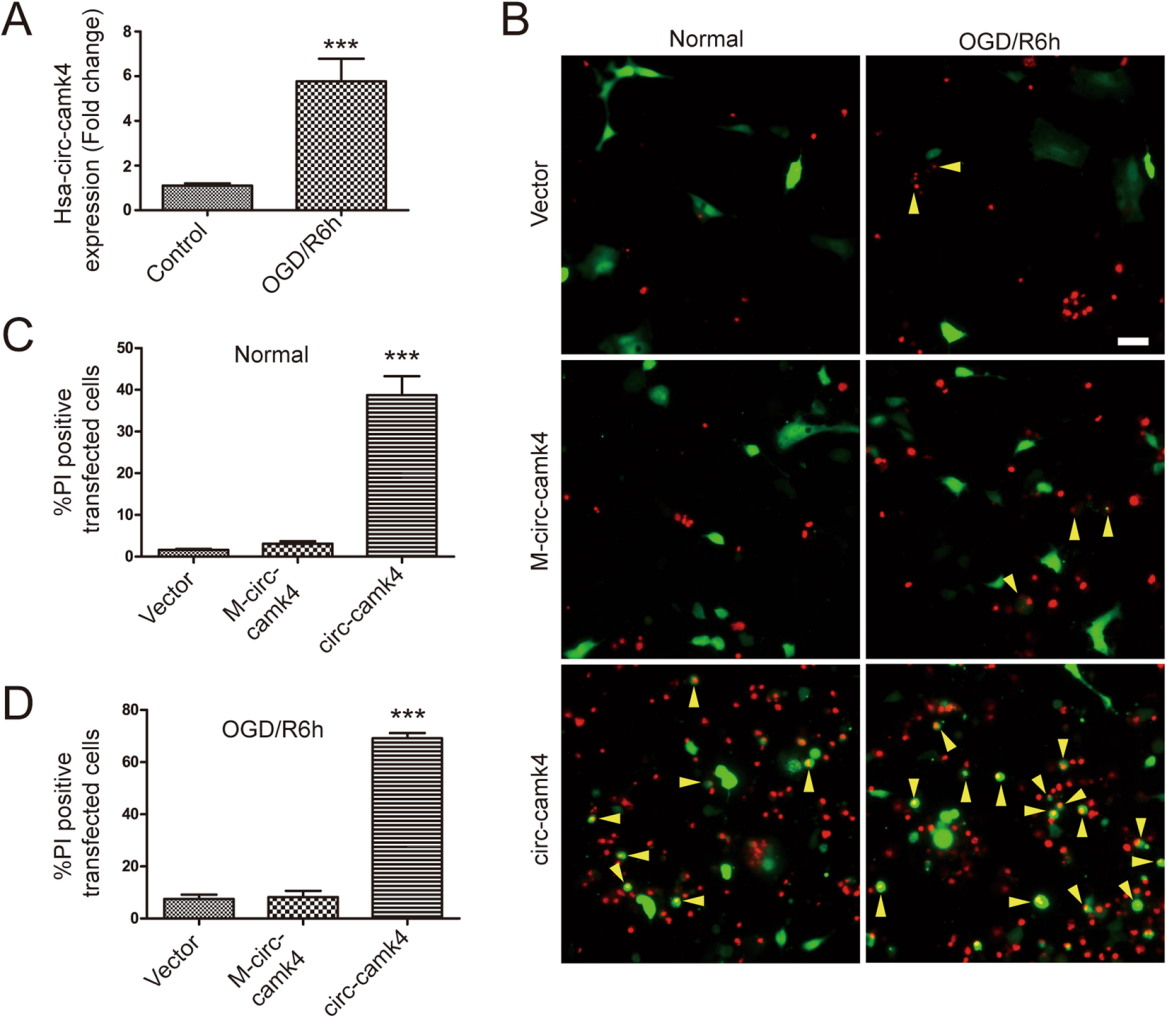
Overexpression of hsa-circ-camk4 increases amounts neuronal cell death *in vitro*. (**A**) RT-PCR analysis of hsa-circ-camk4 expression in SH-SY5Y cells treated with OGD/R. U6 was used as a loading control. (**B**) Representative fluorescence images showing PI- (red) and GFP- (green, indicating transfected cells) positive SH-SY5Y cells that were exposed or not to OGD/R. Arrows indicate dead cells in the population of transfected SH-SY5Y cells. Scale bars = 30 μm. (**C**,**D**) Rate of SH-SY5Y cell death relative to cells transfected with Mis-match circ-camk4 (M-circ-camk4) or circ-camk4-that were or were not exposed to OGD/R. Values in C and D represent the mean ± S.E. ***, p < 0.001 versus the control by Student’s t-test.

### Circ-camk4-targeted miRNA-gene network prediction and annotation

Multiple studies have indicated that circRNA can function by “sponging” miRNAs. Given that miRNAs interact with target sequences via non-stringent pairing, one circRNA was always targeted by multiple miRNAs, which in turn allowed further up regulation of target gene transcription. We first assessed whether the predicted miRNAs could interact with circ-camk4 using the NovelBrain Cloud platform (https://www.novelbrain.com) with a miRNA target prediction pipeline based on TargetScan & miRanda. The top 4 predicted target miRNAs were rno-miR-27a-3p, rno-miR-324-3p, rno-miR-212-3p and rno-miR-504. Meanwhile, 69 target genes of these 4 miRNAs were also identified. The miRNA response elements (MREs) in the circ-camk4 sequence (total 239nt) were determined for the top four miRNAs (Fig. 3A). Rno-miR-212-3p has 2 MREs beginning at 36nt and 213nts from the 5′ end of exon 8, whereas rno-miR-324-3P has 3 MREs (50nt, 134nt, 215nt). There were also 3 MREs forrno-miR-27a-3p (4 nt, 28nt, 218nt) but rno-miR-504 has only one MRE (210nt). The detailed minimum free energy (MFE) of these MREs and the specific sequence alignment were also determined (Fig. 3B). To examine potential circ-camk4 functions, we next constructed a circRNA-miRNA-target gene interaction network based on the predicted listusing Cytoscape where the linkages between circ-camk4 and the top 4 predicted miRNAs and secondary 69 targeted genes were displayed in a network (Fig. 3C). To summarize the functional involvement of these target genes, we carried out a function enrichment analysis using the Novel Brain BioCloud platform (https://www.novelbrain.com). In GO and KEGG analysis of the 69 genes, circ-camk4 was predicted to positively regulate expression of sodium ion transmembrane transporters, GTPase activity, postsynaptic density and interactions among proteins in the synapse and cytoplasm (Fig. 3D, Supp. Figure 2). GO Biological pathway (BP) analysis and KEGG pathway analysis indicated that the pathways most strongly involved in circ-camk4 regulation included those acting at glutamatergic synapses as well as the MAPK signaling pathway, calcium signaling pathway and apoptosis pathway that are involved in response of neuronal cells to I/R injury.

### Validation of the interaction between circ-camk4 and predicted miRNAs

miRNA binding to target genes does not involve perfect pairing. Thus, MREs predicted by computational/bioinformatics tools based on MFE value need to be experimentally validated. To assess whether functional miRNAs are indeed being sponged by circ-camk4, we first synthesized mimics of the four miRNAs having 3′-terminal biotinylation. Meanwhile, a 3′biotinylated circ-camk4 antisense RNA designed to target the split junction and a nonspecific RNA were also synthesized as apositive and negative control, respectively (Supplemental Table 2). The miRNA mimics and control miRNA were transfected into C6 cells and total RNA was isolated 24 hr later. qRT-PCR analysis of the total RNA and subsequent standardization and normalization to GADPH expression and negative control values, respectively, showed that endogenous circ-camk4 expression in C6 cells transfected with circ-camk4-AS was significantly decreased, suggesting that circ-camk4 AS could function as a siRNA upon binding to circ-camk4 (Fig. 4A). Among the four miRNA mimics, three led to significant downregulation of circ-camk4 expression whereas rno-miR-504 did not. This result suggests that these three mimics interacted with circ-camk4. We next performed a biotinylated miRNA pull-down assay followed by RT-PCR of circCamk4 using specific divergent primers to confirm interactions (Fig. 4B). After 40 cycles of PCR amplification, the input cell lysate showed comparable endogenous transcription of circ-camk4 and *gapdh*, whereas the biotin-pull down samples had expression that varied significantly. As in the qRT-PCR analysis, in the pull-down assay rno-miR-504 behaved similarly to the negative control. Meanwhile, the other three miRNAs were pulled down with circ-camk4 AS. These results indicated that miR-504 association with circ-camk4 is very weak, whereas the other three miRNAs were sponged by circ-camk4, consistent the qRT-PCR results (Fig. 4A). qPCR using divergent primers was also performed and the qPCR products were purified and sequenced for confirmation. The results are displayed with splice junction sites indicated (red bars, Fig. 4C) in the results obtained using forward and reverse primers.

### Increase in hsa-circ-camk4 in SH-SY5Y cells exposed to OGD/R and cell death associated with has-circ-camk4 overexpression

We also explored the function of circ-camk4 in the human neuronal cell line SH-SY5Y. We first assessed expression of the human homolog, hsa-circ-camk4, corresponding to exon 4, 5 and 6, using RT-PCR with divergent primer pairs. Subsequent RT-qPCR showed that levels of hsa-circ-camk4 were also increased following OGD/R treatment in a manner consistent with that seen for SH-SY5Y cells (Fig. 5A). These results suggest that findings in rats may be applicable to humans. We next made a has-circCamk4 circular RNA expression vector and prepared endotoxin plasmids for transfection into in SH-SY5Y cells. Two days after transfection, the cells were processed for PI/Hoechst staining. Only when has-circ-camk4 was overexpressed were significant levels of SH-SY5Y cell death seen in comparison to cells transformed with empty vector or mis-matched circ-camk4 plasmid (Fig. 5B,C). After OGD/R treatment, the SH-SY5Y cells overexpressing hsa-circ-camk4 showed even higher amounts of cell death(Fig. 5B,D).

## Discussion

The incidence of ischemic stroke is around 4-fold higher than that for hemorrhagic stroke. However, mechanisms that are activated during very early onset of I/R have been unclear, particularly those associated with noncoding regulatory RNA. A better understanding of these mechanisms that are spontaneously induced in response to I/R injury, and the functional changes that result from this induction changes would be highly valuable for the development of novel treatments for stroke.

Recent evidence showed that circRNAs are important for disease progression via interactions with disease-associated miRNAs. Such interactions have been described as “sponging”. For instance, circRNA competes with miR-7 binding to inhibit the function of this miRNA and in turn affect related signaling pathways in cancer, Alzheimer’s disease, and diabetes^8,15,16^.

To improve our understanding of the pathogenesis of stroke, in this study we used high-throughput sequencing (HTS) methods to detect circRNA expression in cortex tissue from rats subjected to MCAO as an animal model of stroke. Although there are limited data concerning circRNA expression in animal models of stroke, one report did examine the circRNA expression pattern based on HTS of tissues from MCAO rats 96 hours after stroke^17^. In contrast, here we examined MCAO samples by HTS during the very early stages after I/R injury. We found that circRNA expression patterns changed after MCAO in rat cortex tissues after as little as 1 hour of hypoxia followed by 3 hours of reperfusion. Compared with the control group, there were significant differences in the expression of 44 circular RNAs in MCAO tissue. We selected 10 circRNAs for verification by qRT-PCR, which in part confirmed the NGS results. We focused on circCamk4 in subsequent examinations. We assessed circ-camk4 expression in primary cultures of neuronal cells that were exposed to OGD/R to test whether changes in circ-camk4 expression were associated with neuronal rather than brain vascular tissue. The qRT-PCR results showed that changes in circ-camk4 expression following OGD/R treatment of cultured neuronal cells for 3 h were consistent with those observed for tissue from MCAO rats. These results indicate that circ-camk4 expression is indeed affected by ischemia in neuronal cells and suggest that circ-camk4 has a role in pathogenesis of cerebral ischemia injury.

To further examine the possible miRNA-sponging mechanism of circ-camk4 in the MCAO model of cerebral ischemia injury, we performed bioinformatic analyses to generate a circ-camk4-miRNA-target gene interaction network, which will provide a reference for study of the association of differentially expressed circRNAs, miRNAs and their potential targets. We identified miR-27a-3p, miR-324-3p, miR-504, and miR-212-3p as the top four miRNAs predicted to bind to circ-camk4. We experimentally confirmed that at least three of these candidate miRNAs could associate with circ-camk4. A pull-down assay produced results that were agreement with the expression validation and MRE predictions. Previous studies demonstrated that these candidate miRNAs could facilitate neuron survival by regulating expression of genes that have induction of transcription in response to injury. For example, MiR-27a-3p can protect against blood-brain barrier disruption and brain injury by modulating expression of endothelial aquaporin-11^18^. Meanwhile microRNA-27a can suppress expression of the Apaf-1 protein to mitigate hypoxia-induced neuronal apoptosis^19^. For miR-324, miR-324-3p overexpression promoted dysregulation of genes involved in cell death and apoptosis^20^, whereas miR-212 can promote neuronal cell maturation^21^. Up-regulation of circ-camk4 would be predicted to enhance sponging of these miRNAs and in turn promote expression of genes targeted by these miRNAs. Together, these findings indicate that circ-camk4 could be involved in the pathogenesis of cerebral ischemia-reperfusion injury through the promotion of neuronal cell death.

Functional annotation by GO and KEGG analysis indicated that circ-camk4 is likely to affect functions associated with the glutamatergic synapse pathway, MAPK signaling pathway and apoptosis pathway. All of these pathways have already been reported to be involved incerebral I/R injury^22,23^. Thus, circ-camk4 may participate in the pathogenesis of cerebral I/R injury by regulating the activity these pathways.

To validate its functional role in cellular survival, we overexpressed hsa-circ-camk4 (Human circ-camk4 analogue) in SH-SY5Y cells. We observed substantial amounts of SH-SY5Y cell death only when hsa-circ-camk4 was overexpressed. The amount of cell death was even more pronounced in SH-SY5Y cells that overexpressed has-circ-camk4 and were exposed to OGD/R treatment. These results again point to a role for circ-camk4 in the harmful effects I/R, and suggest that circ-camk4 could be a new therapeutic target for stroke. Consistent with our findings, a recent study concerning medulloblastoma also suggested that two types of human circ-camk4 participate in cell apoptosis^24^.

In conclusion, in this study we showed that circ-camk4 expression is spontaneously induced in injured neuronal cells during the early stage of I/R in both the MCAO model and an *in vitro* model with primary neuronal cultures. Furthermore, circ-camk4 could sponge the miRNAs miR-27a-3p, miR-324-3p, and miR-212-3p to enhance expression of their target genes. Functional tests showed that circ-camk4 overexpression in SH-SY5Y cells induced substantial amounts of cell death, indicating a pro-apoptotic role forcirc-camk4 after stroke. Further systematic investigations of the mechanisms associated with circ-camk4 in MCAO-induced neuron injury will be needed to further define the circRNA-miRNA-gene-network. Such findings will increase our understanding of pathways that are invoked during cerebral I/R injury onset and help identify new molecular targets for clinical therapy or diagnostic tools.

## Supplementary information

Supplemental Material.
