# Supplementary material for: Circ-camk4 involved in cerebral ischemia/reperfusion induced neuronal injury
Source: Sci Rep. 2020 Apr 24;10:7012. doi: 10.1038/s41598-020-63686-1 (PMC7181679; doi:10.1038/s41598-020-63686-1)
Supplement: Supplementary file 1 — Supplemental Material. [file 41598_2020_63686_MOESM1_ESM.docx]

**Circ-camk4 involved in cerebral ischemia/reperfusion induced neuronal injury**

Zhao-huan Zhang^1#^, Yue-rong Wang^2#^ Fei Li^1#^, Xiu-ling Liu^2^, Hui Zhang^2^, Zhong-zheng Zhu^3^, Hai Huang^2^, Xiao-hui Xu^1^*

1, School of Preclinical Medicine, Wannan Medical College, Wuhu 241001, China. School of Life Sciences, Shanghai University, Shanghai 200444, China. Department of Neurology,  Changzheng Hospital, 200003, Shanghai, China.

2, School of Life Sciences, Shanghai University, Shanghai, 200444, China.

3, Department of Oncology, Shanghai Tenth People's Hospital, Tongji University School of Medicine, Shanghai 200072, China.

# These authors contribute equally to this work.

*Corresponding to Xiao-hui Xu: xxhxxh@shu.edu.cn

Word Count: 4700.

**Authors Contribution**

Xiao-hui Xu designed experiments; Zhao-huan Zhang carried out the primary neuron culture, SD rat MACO experiments; Yue-rong Wang ,Hui Zhang and Zhao-huan Zhang done the RT-PCR assay experiments. Yue-rong Wang and Xiao-hui Xu analyzed the data. Hui Zhang did the GO and KEEG analysis. Xiu-ling Liu, Zhong-zheng Zhu and Hai Huang did the SH-SY5Y cells transfection and PI assay experiments. Fei Li for RNA-pull down assay and the following RT-PCR. Xiao-hui Xu, Zhao-huan Zhang and Hui Zhang wrote the manuscript.

**Table S1:** Summary of differential expression of circRNAs (Fold change indicated with logFC).

| circ_ID | log2FC(LI/RC) |  | Host gene | |
| --- | --- | --- | --- | --- |
|  | FC | p-value | gene_name | gene_id |
| **Up-regulated differentiation** | | | | |
| rno_circ6505 | 3.008180113 | 0.014413706 | Strn | ENSRNOG00000004806 |
| rno_circ5942 | 2.578259368 | 0.022208097 | Oprk1 | ENSRNOG00000007647 |
| rno_circ2217 | 2.490948098 | 0.007459433 | Phactr1 | ENSRNOG00000014264 |
| rno_circ7641 | 2.459764716 | 0.013538161 | Dock3 | ENSRNOG00000014576 |
| rno_circ2671 | 2.435701539 | 0.00235859 | Camk4 | ENSRNOG00000020478 |
| rno_circ4167 | 2.400834768 | 0.039696281 | Zfr | ENSRNOG00000011627 |
| rno_circ5371 | 2.400834768 | 0.039696281 | Cadps2 | ENSRNOG00000007636 |
| rno_circ6381 | 2.272044126 | 0.045651874 | Ubap2 | ENSRNOG00000052087 |
| rno_circ5421 | 2.272044126 | 0.045651874 | Ppp1r9a | ENSRNOG00000008869 |
| rno_circ8295 | 2.152753524 | 0.023635458 | St6gal2 | ENSRNOG00000046515 |
| rno_circ1527 | 2.005621338 | 0.011618237 | Cobl | ENSRNOG00000004281 |
| rno_circ3634 | 2.002248302 | 0.040093587 | Dlg2 | ENSRNOG00000022635 |
| rno_circ4511 | 1.81323047 | 0.04127558 | Pcdh10 | ENSRNOG00000031974 |
| rno_circ3116 | 1.797358969 | 0.034765337 | Prkcb | ENSRNOG00000012061 |
| rno_circ4226 | 1.589545601 | 0.034456631 | Mctp1 | ENSRNOG00000013282 |
| rno_circ6213 | 1.536551621 | 0.039195221 | Rere | ENSRNOG00000017940 |
| **Down-regulated differentiation** | | | | |
| rno_circ3780 | -3.449636294 | 0.000168571 | Stambpl1 | ENSRNOG00000050224 |
| rno_circ6733 | -2.391591026 | 0.018258403 | Efr3b | ENSRNOG00000012950 |
| rno_circ3730 | -2.391591026 | 0.018258403 | Ttyh1 | ENSRNOG00000032699 |
| rno_circ3376 | -2.316570798 | 0.002510452 | Zranb1 | ENSRNOG00000017294 |
| rno_circ4991 | -2.305263591 | 0.023239034 | Chd6 | ENSRNOG00000016744 |
| rno_circ5115 | -2.264685322 | 0.005545191 | Kiz | ENSRNOG00000025141 |
| rno_circ7545 | -2.221840613 | 0.002831698 | Hmgcll1 | ENSRNOG00000011193 |
| rno_circ6993 | -2.129181175 | 0.008221656 | Rims2 | ENSRNOG00000004201 |
| rno_circ6856 | -2.119504102 | 0.009186799 | Ralgapa1 | ENSRNOG00000046256 |
| rno_circ353 | -2.070508471 | 0.028743676 | Acadvl | ENSRNOG00000018114 |
| rno_circ3753 | -2.057046674 | 0.028123146 | Vti1a | ENSRNOG00000042786 |
| rno_circ260 | -2.010148987 | 0.049335813 | Tlk2 | ENSRNOG00000006285 |
| rno_circ1963 | -2.010148987 | 0.049335813 | Acsl1 | ENSRNOG00000010633 |
| rno_circ7171 | -1.993528797 | 0.033540426 | Tcf20 | ENSRNOG00000009585 |
| rno_circ7294 | -1.988377815 | 0.02695136 | Mapk12 | ENSRNOG00000031233 |
| rno_circ3380 | -1.937689517 | 0.008205229 | Zranb1 | ENSRNOG00000017294 |
| rno_circ2640 | -1.909116881 | 0.020188708 | Htr4 | ENSRNOG00000019134 |
| rno_circ7426 | -1.862140583 | 0.00742541 | Ephb1 | ENSRNOG00000007865 |
| rno_circ229 | -1.81974046 | 0.029463659 | Crebbp | ENSRNOG00000005330 |
| rno_circ7964 | -1.669226967 | 0.024581445 | Tnfrsf21 | ENSRNOG00000011517 |
| rno_circ7326 | -1.642637067 | 0.027317469 | Nav3 | ENSRNOG00000052157 |
| rno_circ6195 | -1.641690159 | 0.019292489 | RGD1310951 | ENSRNOG00000016780 |
| rno_circ3820 | -1.636494297 | 0.03444143 | Sptbn2 | ENSRNOG00000058842 |
| rno_circ6676 | -1.625583522 | 0.045254176 | Ttll5 | ENSRNOG00000009318 |
| rno_circ3379 | -1.625583522 | 0.045254176 | Zranb1 | ENSRNOG00000017294 |
| rno_circ2220 | -1.620715238 | 0.021000858 | Gli3 | ENSRNOG00000014395 |
| rno_circ8138 | -1.618087791 | 0.021106829 | Epb41l3 | ENSRNOG00000016724 |
| rno_circ2462 | -1.562767169 | 0.042739234 | Adarb2 | ENSRNOG00000030775 |


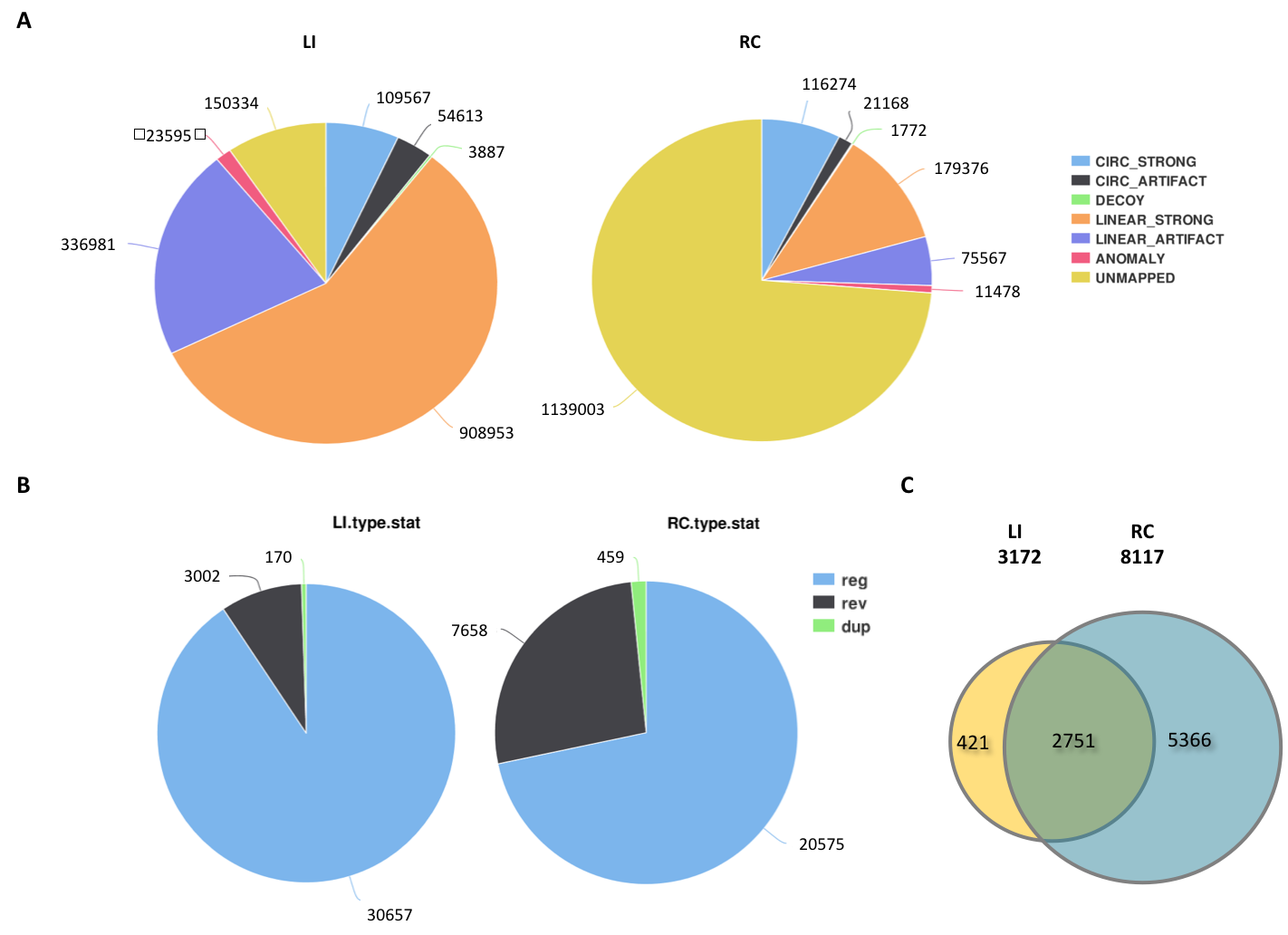


**Figure S1. Statistics of annotation of reads predicted by KNIEF.** A. Pie chart to show types of the pair-end reads annotation on split site by KNIEF, with individual meaning of the aberration as following: CIRC_STRONG, reads mapped to circular split site and p-value > = 0.9 ; CIRC_ARTIFACT, reads mapped to circular split site and p-value < 0.9 ; DECOY, Read1 mapped to the circular split site, read2 does not support the two reads form a circle structure. LINER_STRONG, reads mapped to linear split site and p-value > = 0.9; LINER_ARTIFACT, reads mapped to circular split site and p-value < 0.9 ; ANOMALY, Read1 mapped to the linear split site, read2 does not in line with read1; UNMAPPED, Read1 mapped to the linear cutting site, read2 does not mapped on any exon; B. Pie chart to show types of junctions overall annotated. Abbreviations: reg, regular split junctions; rev, circular split junctions formed by more than 2 exons; dup, circular split junctions formed by a single exon; LI, Left injury side; RC, right control side. C, Venn diagram shows the circular junctions identified in LI and RC, respectively, and the intersection area indicating the junctions identified in both LI and RC.


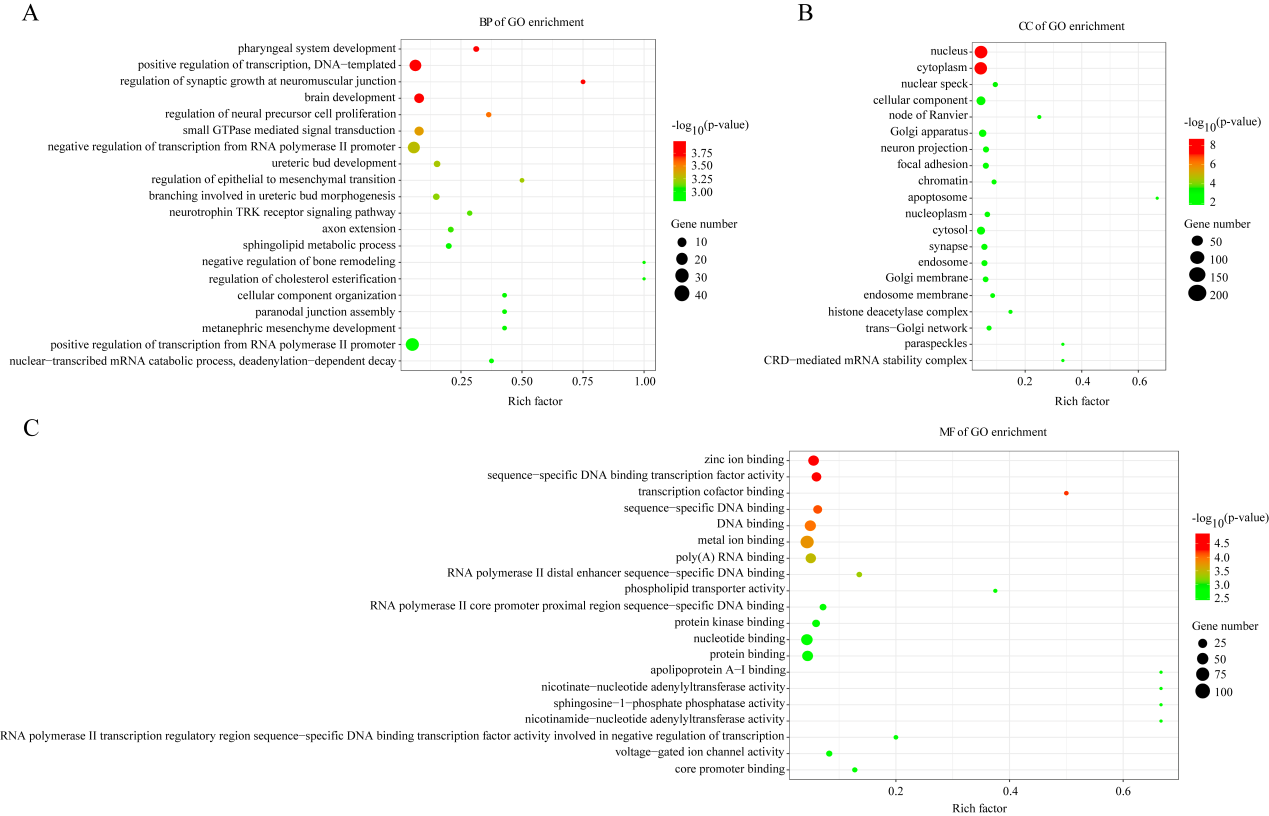


**Figure S2****. The GO annotations for target genes mediated by the miRNAs potentially regulated by circ-camk4.** The GO analysis categorized the mRNAs into different groups under the theme of biological process (BP), cellular component (CC), and molecular function (MF). (A) Scatter plot showing the fold enrichment values of the top 20 most significantly enriched terms for the BP. (B) Scatter plot showing the fold enrichment values of the top 20 most significantly enriched terms for the CC. (C) Scatter plot showing the fold enrichment values of the top 20 most significantly enriched terms for the MF. Rich factor represented the ratio of enriched differential genes to annotated genes in each pathway. The area of each node represented the number of enriched differential genes. The p-value was indicated by different color changes from green to red.


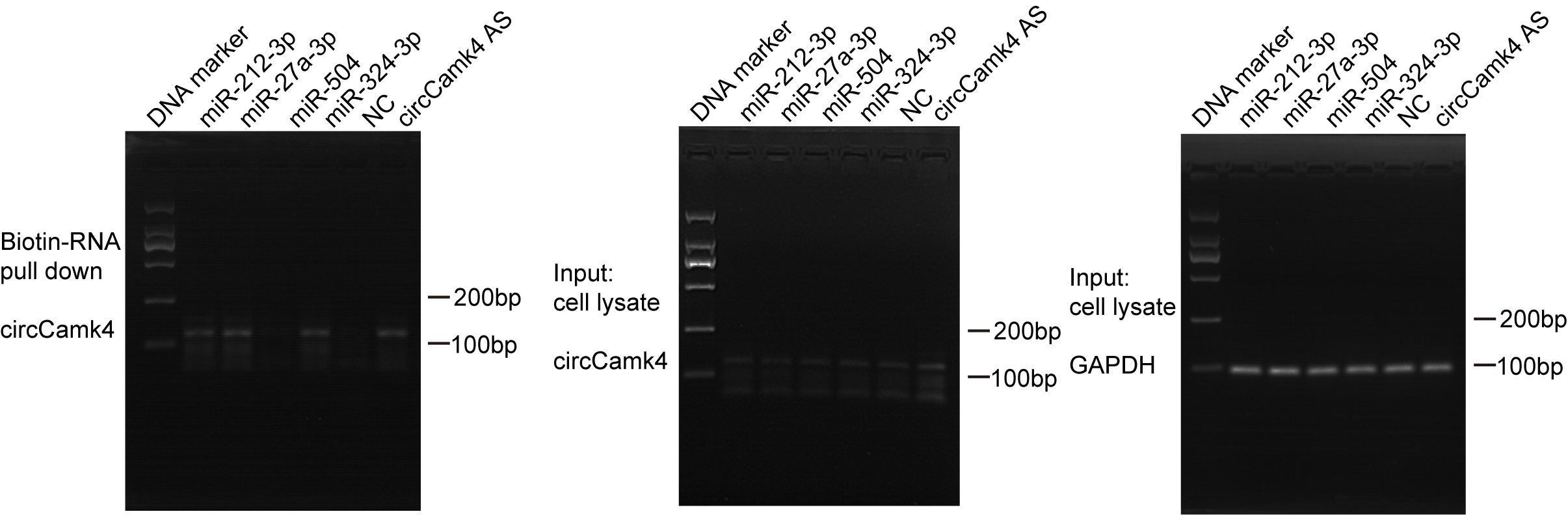


**Figure S3.** **Full gels for Figure 4B.**

RNA oligo sequences:

| rno-miR-212-3p mimics: | Sense strand | UAACAGUCUCCAGUCACGGCCA |
| --- | --- | --- |
|  | Antisense strand: | GCCGUGACUGGAGACUGUUAUU |
| rno-miR-27a-3p mimics: | Sense strand | UUCACAGUGGCUAAGUUCCGC |
|  | Antisense strand | GGAACUUAGCCACUGUGAAUU |
| rno-miR-504 mimics: | Sense strand | AGACCCUGGUCUGCACUCUGUC |
|  | Antisense strand | CAGAGUGCAGACCAGGGUCUUU |
| rno-miR-324-3p mimics: | Sense strand | CCACUGCCCCAGGUGCUGCUGG |
|  | Antisense strand | AGCAGCACCUGGGGCAGUGGUU |
| rno-circ-camk4 AS: | Antisense probe | CUUUCUCCACAAUCCACAGUACCCC |
| Negative control: | Sense strand | UUCUCCGAACGUGUCACGUTT |
|  | Antisense strand | ACGUGACACGUUCGGAGAATT |
